# BLISS: an artificial language for learnability studies

**DOI:** 10.1186/1471-2202-12-S1-P341

**Published:** 2011-07-18

**Authors:** Sahar Pirmoradian, Alessandro Treves

**Affiliations:** 1 Cognitive Neuroscience, SISSA, Trieste, Italy

## 

To explore neurocognitive mechanisms underlying the human language faculty, cognitive scientists use artificial languages to control more precisely the language learning environment and to study selected aspects of natural languages. The aim of the present study is the construction of an artificial Basic Language Incorporating Syntax and Semantics, *BLISS*, which mimics natural languages by possessing a vocabulary, syntax, and semantics. BLISS is generated by a context-free grammar of limited complexity with about 40 production rules, with probabilities that were drawn from the Wall Street Journal corpus. The BLISS vocabulary contains about 150 words which belong to different lexical categories such as noun, verb, adjective, etc., and which were selected from the Shakespeare corpus. Semantics was defined as the dependence of each word on preceding words in the same sentence, purely determined by imposing constraints on word choice during sentence generation. Based on the different algorithms which were applied for the selection of a new word, 4 alternative language models, 3 semantics and one no-semantics, were constructed: *Exponential*, *Subject-Verb*, *Verb-Subject*, and *No-Semantics*.

To measure the effect of introducing semantics to BLISS, the distances between the distributions of consecutive word-pairs in corpora generated by the different language models were measured using Kullback-Leibler (KL) divergence. However, so as to measure purely semantics effect, firstly we attempted to eliminate the effect of word frequency by producing corpora with close word frequencies. Next, looking at the KL-divergences of the distributions of word-pairs, we observed that all the three semantics models are relatively far from No-Semantics one; the Verb-Subject model shows a different kind of dependence between words while the Subject-Verb and Exponential models represent very similar dependence. Furthermore, if we increase the effect of preceding words in word choice, through a parameter in the semantics models, the distances of the semantic models from the no-semantics one considerably increase, underscoring the effect of introducing semantics to the language.

